# The Tumour Suppressor Fhit Protein Activates C-Raf Ubiquitination and Degradation in Human Melanoma Cells by Interacting with Hsp90

**DOI:** 10.3390/biomedicines10102551

**Published:** 2022-10-13

**Authors:** Francesco Paduano, Eugenio Gaudio, Francesco Trapasso

**Affiliations:** 1 Department of Experimental and Clinical Medicine, University Magna Græcia of Catanzaro, 88100 Catanzaro, Italy; 2 Stem Cells and Medical Genetics Units, Tecnologica Research Institute and Marrelli Health, 88900 Crotone, Italy; 3 DTI-Tech, 6500 Bellinzona, Switzerland

**Keywords:** Fragile Histidine Triad Diadenosine Triphosphatase (*FHIT*), heat shock protein 90 (Hsp90), C-Raf, protein–protein interactions (PPIs), melanoma

## Abstract

Fhit protein expression is reduced in the majority of human tumors; moreover, its restoration both triggers apoptosis of cancer cells and suppresses tumor formation in a large number of preclinical models of cancers. In the following study, we observed that Fhit expression is significantly reduced in human melanoma cells, and their in vivo growth is blocked by a recombinant adenovirus carrying the *FHIT* gene. Importantly, we found here that Fhit physically interacts with Hsp90. Since Hsp90 is a chaperone with a crucial function in the conformational maturation and stabilization of C-Raf, we also investigated whether Fhit could interfere with the Hsp90/C-Raf protein complex in melanoma. Interestingly, the administration of the Hsp90 inhibitor 17-AAG, in combination with Fhit protein overexpression in melanoma cells, reacts synergistically to increase C-Raf ubiquitination and degradation. These data reveal Hsp90 as a novel interactor of Fhit and suggest that *FHIT* activity restoration could represent a helpful strategy for suppressing the oncogenic C-Raf pathway in the therapy of human melanoma.

## 1. Introduction

The *FHIT* gene maps on chromosome 3p14.2 and encompasses the most active fragile site in the human genome, known as FRA3B. The t(3;8) (p14.2-q24) translocation, which leads to impaired Fhit protein expression, was discovered in 1996 in a family affected by renal cancer [1]. Although early in vitro studies showed that Fhit is an enzyme able to hydrolyse diadenosine triphosphates, which are produced in cells in response to stress [2], its activity as a tumor-suppressing protein—although frequently assessed both in experimental and human tumorigenesis [3,4]—remained obscure for a long time since no protein partners were proven for a long time after its discovery. Only more than a decade after its identification has our group identified a list of Fhit candidate partners, including among them, FRDX, HSP10 [4], and annexin A4 (A4) [5]. We also identified a short 7-aminoacids peptide from the Fhit protein that is still able to bind A4 and sensitize lung cancer cells to paclitaxel, thus recapitulating the activity of the full-length Fhit protein on A4 [6,7].

Even though some investigations reported that *FHIT* hypermethylation, a common mechanism of *FHIT* inactivation, in uveal melanoma is a very rare event [8,9], the role of Fhit protein in human melanogenesis was never deeply investigated.

Melanoma is considered one of the most aggressive human cancers and one of the most difficult cancers to treat due to its resistance to current therapies [10,11]. Despite the advances in metastatic melanoma treatment, including target therapy and immunotherapy, this disease is very problematic to treat and still remains a lethal type of cancer [12,13]. For example, it has been shown that 5-year survival after diagnosis for metastatic melanoma is around 50% when treated with combination immunotherapy and 33% with combination BRAF/MEK targeted therapy [13].

Melanoma harbors multiple genetic alterations that corrupt biological pathways controlling cell growth, differentiation, survival, and metabolic reprogramming [11,14]. Among these pathways, the MAPK cascade, including RAS, RAF, MEK, and ERK, as well as other signaling pathways, such as WNT and PI3K-AKT, have been shown to be frequently mutated in melanoma [11,14]. Genetic alterations in *BRAF* and *NRAS* genes are very common in primary melanomas, whereas other mutations are involved in *NRAS*, *TP53*, *TERT*, *CDKN2A*, and *PTEN* genes [15]. For example, the *BRAF* V600E is the most frequent genetic cause of melanoma, which is responsible for the increased proliferation and metabolic reprogramming of melanoma cells [11,16].

Although today there are several successful melanoma therapies, such as approaches that primarily suppress the BRAF oncoprotein pathway [13], it is very important to investigate new therapeutic approaches and molecular targets. This happens because it has been shown that 15–20% of primary melanomas can become resistant to drugs, such as BRAF/MEK inhibitors [14,15].

Here, we demonstrated that Fhit negatively modulates C-Raf expression in melanoma cells by interacting with Hsp90. Moreover, the restoration of Fhit expression in Fhit-negative melanoma cells triggers apoptosis and blocks tumor formation in vivo. These results suggest a further pathway to be explored for the generation of novel tools for the therapeutic targeting of human melanoma.

## 2. Materials and Methods

### 2.1. Transfection and Cell Cultures

Cell cultures and experiments with transfections were executed as previously described [5]. Briefly, melanoma cell lines were cultivated at 37 °C and 5% CO_2_. HEK293 cells were used for the construction and amplification of the recombinant adenovirus vectors.

### 2.2. Immunoblotting Analysis

Immunoblotting analyses were performed as previously described [17,18,19,20]. Briefly, enriched plasma membrane proteins were carried out using a specific kit (Bio-Rad, Pleasanton, CA, USA). These proteins were then analyzed on polyacrylamide gels and subsequently moved to membranes of nitrocellulose (Bio-Rad). Subsequently, blocked membranes were incubated with primary antibodies and detected by the specific secondary antibodies. Co-IP experiments were carried out as previously described [20]. Briefly, total lysates were incubated with the anti-Hsp90 antibody at 4 °C O.N. Subsequently, antibodies or beads were washed and boiled in a buffer containing SDS, and then proteins were analyzed on polyacrylamide gels (4–20%) with or without the use of the crosslinking agent known as [(dithiobis(succinimidylpropionate), or DSP]).

### 2.3. Protein Interaction Evaluation

Co-immunoprecipitation (Co-IP) assays were performed as previously described [20]. Briefly, enriched plasma membrane proteins, with or without the addition of DSP, were incubated with magnetic agarose beads (NIN-TA, Qiagen, Washington, USA), and the resulting interacting proteins were obtained.

### 2.4. Construction of Recombinant Adenoviral Vectors

Ad*FHIT* (an adenovirus carrying the *FHIT* cDNA) and Ad*FHIT*-His6 (an adenovirus carrying the *FHIT* cDNA with a His_6_ epitope tag) were obtained as previously described [5]. Detailed methods are described in the Supplementary Materials and Methods.

### 2.5. In-Vitro Cell Growth Evaluation

Melanoma cells, seeded on plates, were infected with an adenovirus carrying *FHIT* cDNA (Ad*FHIT*), *GFP* cDNA (Ad*GFP*), or a control (mock-infected). Subsequently, cells were counted at 24 h intervals.

### 2.6. Proteomic Experiments

Mass spectrometry studies, as well as a digestion and MALDI analysis, LTQ, and protein identification, were carried out as previously described [21]. Detailed methods are described in the Supplementary Materials.

### 2.7. TUNEL Assay

Tunel assays were performed as previously described [5]. Briefly, apoptosis of the A549 cells was measured by a TUNEL assay (Boehringer/Roche, Indianapolis, IN, USA).

### 2.8. Ubiquitination Assays

Protein lysates were incubated with O.N. with an antibody against C-Raf (Cell Signaling, Beverly, MA, USA) or AG/agarose beads and subsequently separated on polyacrylamide gels (4–20%). Finally, obtained membranes of nitrocellulose were immunoblotted with an antibody against HA-HRP to detect Ub-HA (ubiquitin) and antibodies against Fhit and C-Raf (Cell Signaling, Beverly, MA, USA).

### 2.9. Immunohistochemistry

Colo38 cells on the slide were hydrated in PBS for 5 min. Next, slides were soaked in TBS with tween for 5 min, quenched with 0.03% H_2_O_2_ for 5 min, and then rinsed with TBST. The primary antibody, C-Raf (LifeSpan BioSciences Seattle, WA, USA), was diluted at 1:1000 and then incubated for 1 h at RT. The color reaction was developed with Red Vulcan for 10 min. Lastly, slides were stained with Mayer’s Haematoxylin and mounted.

### 2.10. Animal Studies

Animal studies were carried out under the guidelines validated by the animal facility of Ohio State University. Tumors were evaluated as previously described [5]. Briefly, Colo38 cells were injected into the right flanks of female nude mice (nu/nu) at 6 weeks of age. The following formula estimated tumor volumes: V (in mm^3^) = A × B2/2, in which A was the measure of the largest diameter and B was the measure of the perpendicular diameter.

### 2.11. Statistics

Results are expressed as the mean ± standard deviation of three separate experiments. The significance of the differences between groups was evaluated using Student’s *t*-tests or one-way ANOVAs. Analysis was conducted using GraphPad Prism software (San Diego, CA, USA), and differences were considered significant if *p* < 0.05.

Values were represented as means ± SD derived from 3 independent experiments. Differences among groups were calculated using Student’s *t*-tests, and significance was determined at *p* < 0.05.

## 3. Results

### 3.1. Identification of Hsp90 as a Candidate Fhit Protein Interactor

As described in our previously published study, Hsp90 was identified through mass spectrometry as a putative partner of the Fhit protein [5]. We analyzed the physical interaction between these two proteins as described below.

### 3.2. Fhit Interacts with Hsp90

**Figure 1:**
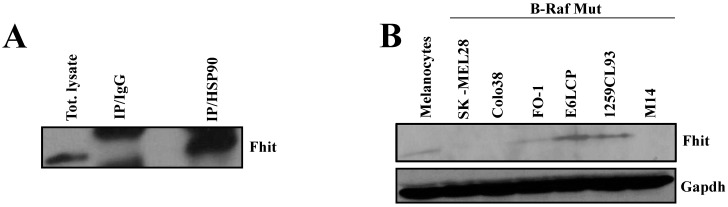
Hsp90 interacts with Fhit. (**A**) Proteins derived from Colo38 were immunoprecipitated using an agarose-conjugated antibody against IgG or Hsp90, and immunoprecipitates were separated on SDS-PAGE and subsequently blotted with Fhit and Hsp90 antibodies. (**B**) Fhit protein expression was evaluated with normal melanocyte and melanoma cancer cells via W.B. with an antibody against Fhit. Gapdh and B-actin were used as loading controls.

To determine whether Fhit and Hsp90 physically interacted, we performed a co-immunoprecipitation experiment. As shown in Figure 1A, Colo38 melanoma cells were infected with Ad-*FHIT* and subsequently treated with DSP. The resultant proteins were immunoprecipitated with protein A/G agarose and IgG or anti-Hsp90 antibodies and, finally, were tested using Western blotting. These results confirmed that the Fhit protein interacts with Hsp90 (Figure 1A). Similarly, the interaction between Fhit and Hsp90 was further confirmed through immunoprecipitation experiments, where cells were infected with an adenovirus carrying *FHIT* cDNA modified with a sequence encoding a His_6_ epitope tag (Ad*FHIT*-His_6_) at its 3′ end (data not shown).

We subsequently analyzed Fhit protein expression in several melanoma cell lines, including SK-Mel28, FO-1, E6LCP, 1259CL93, M14, Hs294T, 1106, and Colo38 having mutations in *B-RAF* and WM13066, MeWo and CHL-1 having *B-RAF* WT, as well as normal melanocytes via Western blotting (Figure 1B and data not shown). Results indicated that the levels of Fhit expression were lower in some melanoma cell lines with respect to their normal counterpart.

### 3.3. Fhit Affects C-Raf Expression in Human Melanoma

**Figure 2:**
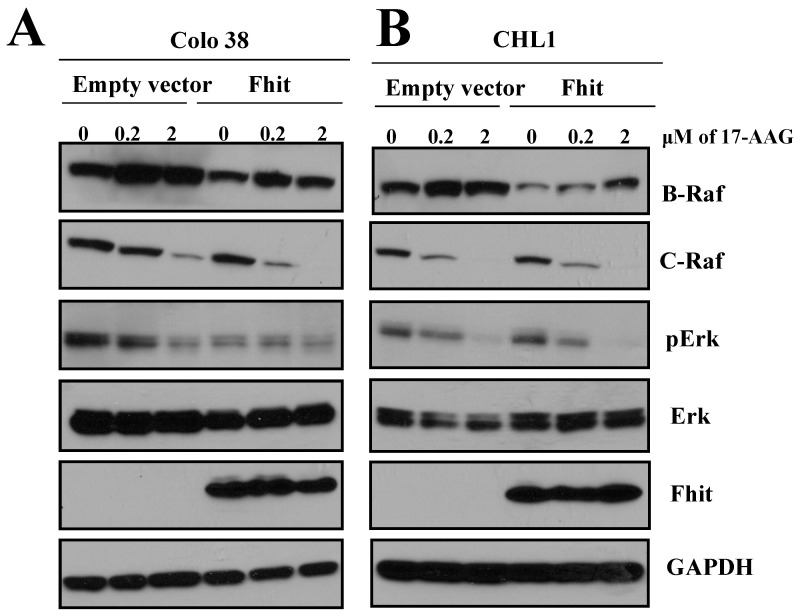
Fhit affects C-Raf expression through Hsp90. (**A**,**B**) Colo38 and CHL-1 melanoma cell lines were transfected with an empty vector or Fhit and treated with 17-AAG or DMSO at the indicated concentration for 24 hrs. Then, cell lysate was separated on SDS-PAGE gel and blotted with B-Raf, C-Raf, p-ERK, ERK, Fhit, and GAPDH antibodies.

Since it is well known that Hsp90 regulates C-Raf folding, we sought to determine whether Fhit affected C-Raf expression through Hsp90 via Western blotting. To this end, melanoma cell lines, Colo38 (*B-RAF* V600E) and CHL-1 (*B-RAF* wild-type), were mock- and *FHIT*-transfected and treated with the inhibitor of Hsp90 known as 17-allylamino-17-dimethoxy geldanamycin (17-AAG) for 24 hrs (Figure 2A,B). Results showed that the C-Raf and p-ERK expressions were downregulated through Fhit expression (Figure 2A,B; lane 4 vs. lane 1) and 17-AAG treatment (lanes 5, 6 vs. lanes 2, 3). Hsp90 weakly regulates B-Raf expression, and for this reason, 17-AAG does not affect much B-Raf expression. It is probable that B-Raf expression is increased as a compensatory effect of C-Raf downregulation. Significantly, since an exogenous expression of Fhit was responsible for the reduced expression of C-Raf, we performed a ubiquitination assay. To this end, Colo38 cells were transfected with *Ub-HA* and *FHIT* expression vectors. After 48 h of transfection, cells were exposed to 17-AAG and the proteasome inhibitor, MG132, at a concentration of 10 μM for an additional three hours. Subsequently, after the lysis of the cells, the resultant proteins were immunoprecipitated against C-Raf with an antibody, followed by immunoblotting with an antibody against the HA epitope. Results revealed that the expression of Fhit, combined with additional treatment with 17-AAG, resulted in C-Raf ubiquitination (data not shown). These data suggest that Fhit expression disturbs Hsp90’s activity and enhances 17-AAG effects by producing, as the read-out, the severe ubiquitination of C-Raf. Significantly, melanoma cells shift their dependency from B-Raf to C-Raf and vice versa; we were not able to see any regulation or ubiquitination of B-Raf by Fhit alone or in combination with 17-AAG.

### 3.4. Restoration of Fhit Expression Affects Proliferation of Melanoma Cells

**Figure 3:**
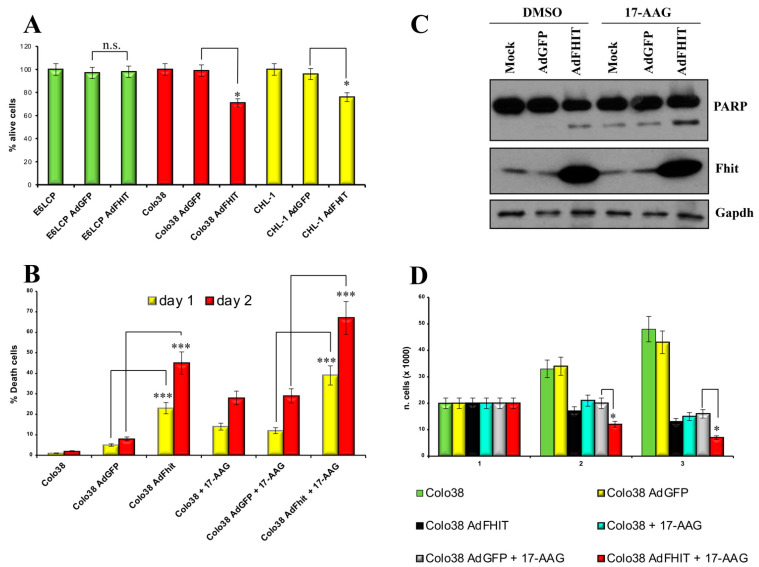
Restoration of Fhit expression reduces cell proliferation and induces apoptosis in human melanoma. (**A**) E6LCP, Colo38 and CHL1, Ad*GFP*, Ad*FHIT,* or mock-infected cells were analyzed using MTS assays (Student’s *t*-test, * *p* < 0.05; n.s., not significant). (**B**) Colo38 Ad*FHIT* and Ad*GFP,* untreated or treated with 17-AAG at 2 μM, were analyzed using a TUNEL assay (one-way ANOVA, *** *p* < 0.001). (**C**) Cell lysates from the experiment described in B were blotted with antibodies against Fhit, Parp, and Gapdh. (**D**) Colo38 Ad*GFP*, Ad*FHIT,* or mock-infected cells, untreated or treated with 17-AAG at 2 μM, were counted, and cell growth curves were calculated. Results show means ± SD from 3 independent experiments (Student’s *t*-test, * *p* < 0.05).

To determine whether the *FHIT* gene could be used as a therapeutic agent, we constructed a replication-defective adenovirus encompassing the *FHIT* gene and a *GFP* reporter gene as previously described [22]. Colo38 and CHL-1 cells were infected with Ad*FHIT* or Ad*GFP*, and levels of adenovirus infection were observed using confocal microscopy 48–72 h after infection. Subsequently, exogenous Fhit protein expression was evaluated through Western blotting. Data showed that the restoration of Fhit expression significantly affected the proliferation of melanoma cells, such as Colo38 and CHL-1 (Figure 3A).

### 3.5. Restoration of Fhit Induces Apoptosis in Human Melanoma

We also examined the role of Fhit expression on the proliferation of E6LCP and Colo38 melanoma cells through MTS. Interestingly, Colo38 melanoma cells, which do not express Fhit endogenously, were strongly affected, in terms of proliferation, by the re-expression of adenovirus-mediated Fhit. On the contrary, E6LCP melanoma cells expressing Fhit endogenously were slightly affected by an over-expression of Fhit (Figure 3A). In addition, Colo38 melanoma cells were analyzed using TUNEL assays to evaluate the apoptotic effects of Fhit gene therapy alone or combined with 17-AAG (Figure 3B). Results showed that the exogenous expression of Fhit induces apoptosis in human melanoma Colo38, and that the levels of apoptosis were further increased when using 17-AAG on both days 1 and 2 after treatment.

To corroborate the effect of Ad*FHIT* on melanoma cell proliferation, we treated Colo38 with Ad*FHIT* and Ad*GFP*, alone or in combination with 17-AAG (Figure 3C).

Results showed that the proliferation of melanoma Ad*FHIT*-infected cells was significantly reduced after 3 days of treatment. Importantly, Colo38 proliferation levels were further decreased when using 17-AAG in combination with Ad*FHIT* (Figure 3D).

### 3.6. Fhit Regulates C-Raf Expression in Melanoma Cells

**Figure 4:**
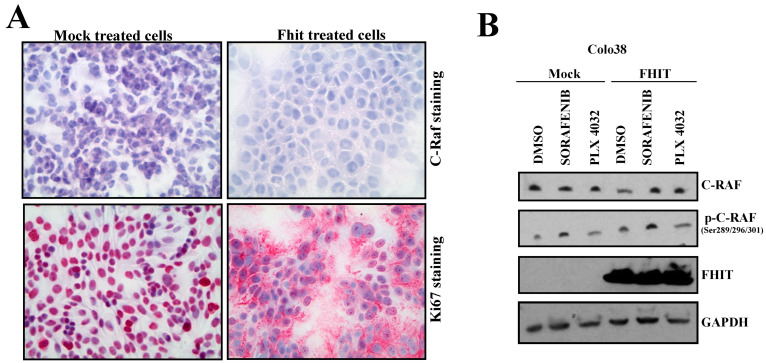
Fhit regulates C-Raf expression in melanoma cells. (**A**) C-Raf, Ki67, and H&E were analyzed through immunohistochemistry in mock- and *FHIT*-transfected Colo38 melanoma cells. (**B**) Colo38 melanoma cells, mock- or *FHIT*-transfected, were treated with DMSO, Sorafenib, and PLX4032. Then, cell lysates were immunoblotted with C-Raf, p-C-RAF, FHIT, and GAPDH antibodies.

Subsequently, C-Raf protein expressions were assessed by immunohistochemistry on mock- and Fhit-transfected Colo38 melanoma cells (Figure 4A). Immunohistochemistry assays demonstrated a significant reduction in C-Raf protein expression when Fhit was overexpressed by transfection, which confirmed our Western blotting data.

Using mock- and *FHIT*-transfected Colo38 melanoma cells, subsequently treated with DMSO, Sorafenib (an inhibitor of C-Raf and mutated B-Raf), and PLX4032 (an inhibitor of mutated B-Raf), we observed the inactivating phosphorylation of C-Raf on Ser 289/296/301 was relevant in all *FHIT*-transfected conditions. An additive effect between Fhit and the kinase inhibitor drug treatments was detected (Figure 4B).

### 3.7. Tumour Suppressor Activity of Fhit in Melanoma Xenograft

**Figure 5:**
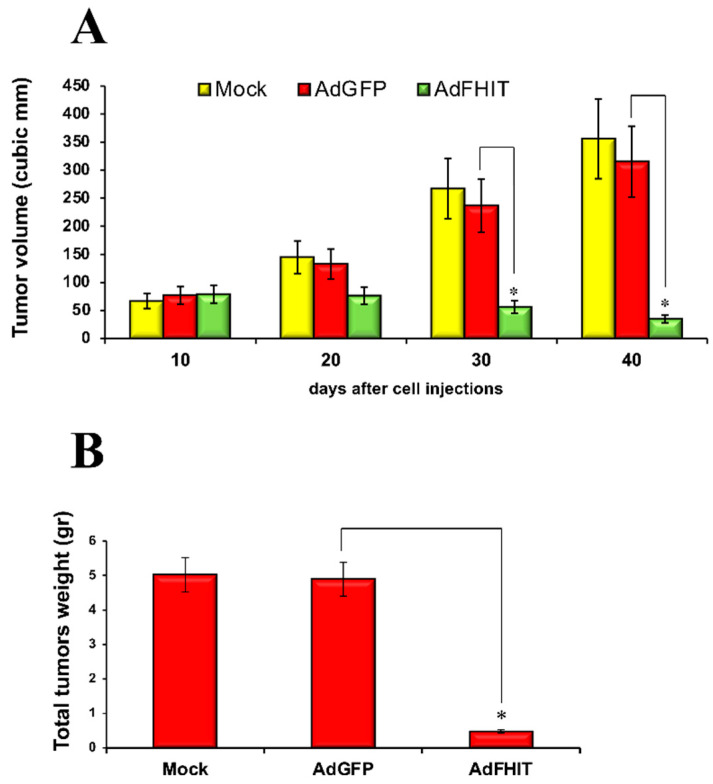
Tumor suppressor activity of Fhit in melanoma xenograft. (**A**) Tumor volumes at 10, 20, 30, and 40 days for the mock, Ad*GFP,* and Ad*FHIT* groups of mice (5 mice/group). Overall, 10 × 10^6^ Colo38 cells were pre-infected with Ad*FHIT* or Ad*GFP* for 12 h and then injected subcutaneously into nude mice (5 mice for the group). Tumors were measured every 10 days, as reported in the graph (Student’s *t*-test, * *p* < 0.05). (**B**) Tumors were extracted and weighed after 40 days (Student’s *t*-test, * *p* < 0.05).

Finally, we checked the effects of Fhit in vivo in a preclinical model of melanoma. To this end, Colo38 cells pre-infected with Ad*FHIT* at MOI 50 were injected into the flank of nude mice. After ten days of injection, tumors of around 8 mm in diameter were observed in some groups of mice. Results showed that tumors appeared both in the control groups of mice injected with mock-infected cells or AdGFP-infected cells. Importantly, xenograft tumors treated with Ad*FHIT* displayed a significant reduction in volume and weight with respect to the control group (Figure 5A,B).

## 4. Discussion

In this study, by using adenovirus vectors and a proteomic approach, we identified Hsp90 as a novel interactor of Fhit. The methodological approach used in this study was previously successfully used to isolate and characterize soluble Fhit protein complexes, including Hsp10/Hsp60, ferredoxin reductase [4], and annexin 4 [5]. Since it is well known that Fhit is downregulated in most cancer cell lines [1,2], we also extended this investigation to melanoma cell lines, where it appears mostly downregulated. Moreover, to clarify Fhit tumor suppressor activity in melanoma, we studied Fhit protein complexes using our validated proteomic approach [5,21].

In this study, Hsp90 was recognized as a candidate Fhit partner, and co-immunoprecipitation experiments confirmed their interaction. Importantly, Hsp90 is crucial for the folding of many oncogenic proteins [23]. Hsp90 is a chaperone, playing a crucial function in the folding and stabilizing of many signaling oncoproteins, and is essential for the correct folding of C-Raf [23,24,25,26]. It was shown that the inhibition of the heat shock protein 90 (Hsp90) function with geldanamycin, or its derived 17-allylamino-17-demethoxygeldanamycin (17-AAG), effectively causes the ubiquitination of C-Raf in the proteasome [27]. Significantly, the folding of Raf family members, such as A-Raf, B-Raf, and C-Raf, is mediated by Hsp90 and other players, such as cdc37, Hop, and Hsp70. These complexes fold and stabilize several proteins involved in cancer pathogenesis [28]. C-Raf and its related RAS/RAF/MEK pathway are hyperactivated in 30% of human cancers and most melanomas. Notably, the B-Raf gene was observed to be mutated in 50% of human melanomas. A strong proliferation and survival rate characterized the cancer cells with the V600E B-Raf allele (B-Raf ^V600E^), and these cells are also more sensitive to 17-AAG than wild-type protein [29].

Since C-Raf is a kinase overexpressed in melanoma, and its folding is under Hsp90 control [30], we studied the role of Fhit in this complex. Here, we observed that the direct interaction between Fhit and Hsp90 can affect Hsp90 activity and, subsequently, C-Raf expression. We demonstrated that C-Raf was ubiquitinated under the combined treatment of Fhit expression and 17-AAG much more than under a single treatment.

Melanoma is one of the most aggressive human tumors, and its incidence and mortality rate have increased worldwide in the last five years [31]. We measured the protein expression levels of Fhit in a panel of twelve melanoma human cell lines and demonstrated that Fhit expression in such cells was reduced or undetectable compared to primary normal human melanocytes. Immunohistochemistry assays showed a significant reduction in C-Raf in Colo38 cells when Fhit was overexpressed by transfection.

To assess *FHIT* activity as a therapeutic gene in melanoma, we generated a recombinant adenovirus encompassing the human cDNA of *FHIT* (Ad*FHIT*). Our experiments established that Fhit has therapeutic activity, both in vitro and in vivo, on melanoma cells. Fhit-negative Colo38 melanoma cells transduced with Ad*FHIT* showed a significantly reduced proliferation rate and underwent programmed cell death. The in vivo investigation of Ad*FHIT* as a therapeutic gene in melanoma was further assessed on a melanoma preclinical xenograft model established by subcutaneously injecting human melanoma cells in nude mice. In vivo, *FHIT* expression effectively reduced tumor growth compared to the control.

Our research group has a long history of studies on the role of Fhit in tumorigenesis [7,32] and, more recently, on the identification of Fhit partners [4,5,6]. All identified Fhit interactors partly shared among different types of cancer cell lines, including lung cancer and melanoma, were investigated in order to both uncover their functional role in cancer and to try to propose anticancer solutions with novel mechanisms of action. In this study, our data suggest that the re-expression of Fhit and its interaction with Hsp90 exerts an anti-tumor effect on human melanomas by modulating the C-Raf activity, especially when combined with Hsp90 inhibitors. These findings may provide hints for therapeutic approaches that could be beneficial for improving the treatment of human melanoma.

## Data Availability

The data can be requested by emailing the corresponding authors.

## Supplementary Materials

The following supporting information can be downloaded at: https://www.mdpi.com/article/10.3390/biomedicines10102551/s1.
