# Supplementary material for: The Tumour Suppressor Fhit Protein Activates C-Raf Ubiquitination and Degradation in Human Melanoma Cells by Interacting with Hsp90
Source: Biomedicines. 2022 Oct 13;10(10):2551. doi: 10.3390/biomedicines10102551 (PMC9599145; doi:10.3390/biomedicines10102551)

Supplementary materials and methods

Mass spectrometry studies

Protein pellets were solubilized and digested by trypsin. Protein constituents were identified by liquid chromatography tandem mass spectrometry (LC-MS/MS). Inspection of LC-MS/MS data was undertaken to assess the exclusive presence of mass peaks belonging to candidate partner proteins in samples from cells infected with Ad*FHIT*-His6.

Digestion and MALDI Analysis

Immunoprecipitated protein complexes were digested with sequencing grade trypsin from Promega (Madison WI) using the Multiscreen Solvinert Filter Plates from Millipore (Bedford, MA). Briefly, the complexes were incubated with dithiothreitol (DTT) solution (25 mM in 100 mM ammonium bicarbonate) for 30 min before the addition of 55 mM Iodoacetamide in 100 mM ammonium bicarbonate solution. Iodoacetamide was incubated with the protein complexes in the dark for 30 min before removal. Enzymatic digestion was carried out with trypsin (12.5 ng/μL) for 18 h at 37 ^{o}C. The digestion was stopped with the addition of 0.5% trifluoroacetic acid TFA. The MS analysis was immediately performed to ensure high-quality tryptic peptides with minimal non-specific peptides.

Mass Spectrometry, LTQ

Capillary-liquid chromatography-nanospray tandem mass spectrometry (Nano-LC/MS/MS) was performed on a Thermo Finnigan LTQ mass spectrometer equipped with a nanospray source operated in positive ion mode. The LC system was an UltiMate™ 3000 system from Dionex (Sunnyvale, CA). Solvent A was water containing 50mM acetic acid, and the solvent B was acetonitrile. 5 ml of each sample was first injected onto the m-Precolumn Cartridge (Dionex, Sunnyvale, CA) and washed with 50 mM acetic acid. The injector port was switched to inject, and the peptides were eluted off the trap onto the column. A 5 cm 75 mm ID ProteoPep II C18 column (New Objective, Inc. Woburn, MA) packed directly in the nanospray tip was used for chromatographic separations. Peptides were eluted directly off the column into the LTQ system using a gradient of 2-80%B over 45 min, with a flow rate of 300 ml/min. The total run time was 65 min. The MS/MS was acquired according to standard conditions established in the lab. Briefly, a nanospray source operated with a spray voltage of 3 KV and a capillary temperature of 200P^{o}PC. The scan sequence of the mass spectrometer was based on the TopTen™ method; the analysis was programmed for a full scan recorded between 350 – 2000 Da, and a MS/MS scan to generate product ion spectra to determine the amino acid sequence in consecutive instrument scans of the ten most abundant peak in the spectrum. The CID fragmentation energy was set to 35%.

Dynamic exclusion was enabled with a repeat count of 2 within 10 sec, a mass list size of 200, and an exclusion duration of 350 sec, the low mass width was 0.5 and the high mass width was 1.5.

Protein identification

The RAW data files collected on the mass spectrometer were converted to mzXML and MGF files by use of MassMatrix data conversion tools (version 1.3, http://www.massmatrix.net/download). For low mass accuracy data, tandem MS spectra that were not derived from singly charged precursor ions were considered as both doubly and triply charged precursors. The resulting MGF files were searched using Mascot Daemon by Matrix Science version 2.2.2 (Boston, MA), and the database was searched against the full SwissProt database version 57.5 (471472 sequences; 167326533 residues) or NCBI database version 20091013 (9873339 sequences; 3367482728 residues). The mass accuracy of the precursor ions was set to 2.0 Da, given that the data was acquired on an ion trap mass analyzer and the fragment mass accuracy was set to 0.5 Da. Considered modifications (variable) were methionine oxidation and carbamidomethyl cysteine. Two missed cleavages for the enzyme were permitted. A decoy database was searched to determine the false discovery rate (FDR), peptides were filtered according to the FDR and proteins identified required bold red peptides. Protein identifications were checked manually, and proteins with a Mascot score of 50 or higher with a minimum of two unique peptides from one protein having a *-b* or *-y* ion sequence tag of five residues or better were accepted.

Generation of recombinant adenovirus

The recombinant adenovirus carrying the wild-type FHIT cDNA (AdFHIT) was prepared as previously described [4] Briefly, a His-tagged FHIT cDNA was generated by PCR with the following oligonucleotides: 5′-ACgTggATCCCTgTgAgg ACATgTCgTTCAgATTTggC-3′(forward) and 5′-TTgTgg ATCCTTATCAgTgATggTgATggTgATgCgATCCTCTC TgAAAgTAgACCCgCAg-3′. These primers were designed with a BamHI restriction site for subcloning into the transfer vector pAdenoVator-CMV5-IRES-GFP. The AdFHIT-His6 was generated with the AdenoVatorTM kit (Qbiogene, Carlsbad, CA), following the manufacturer's procedure. AdGFP, used as a control, was purchased from Qbiogene.

Original image of western blot in Figure 1A

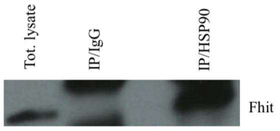

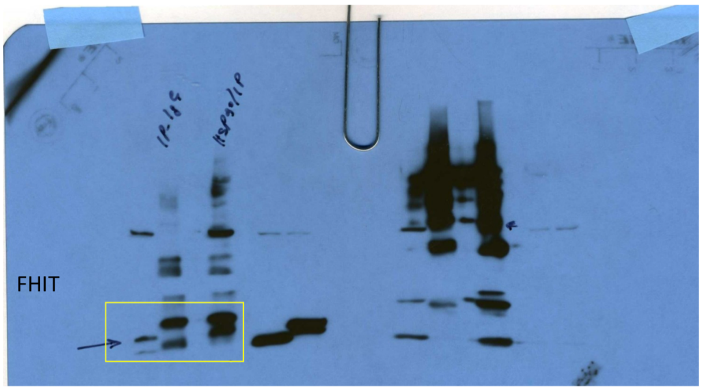

Supplement: Supplementary file 1 [file biomedicines-10-02551-s001.zip › biomedicines-1922471-supplementary.pdf]
